# Construction and Validation of a Risk Prediction Model for Early Severe Intraventricular Hemorrhage in Very Low Birth Weight Infants

**DOI:** 10.1002/kjm2.70037

**Published:** 2025-05-19

**Authors:** Fei Shen, Jie Xu, Hui Rong, Jing Zhang, Yang Yang, Xian‐Wen Li

**Affiliations:** ^1^ Department of Neonatology Children's Hospital of Nanjing Medical University Nanjing People's Republic of China; ^2^ School of Nursing Nanjing Medical University Nanjing People's Republic of China

**Keywords:** intraventricular hemorrhage, neonate, neurodevelopmental disorders, risk prediction model, very low birth weight

## Abstract

In the past several years, prediction models for severe intraventricular hemorrhage (IVH) in premature infants have emerged. However, few models have considered the importance of predictors related to the clinical course and hemostatic profile in predicting the risk of hemorrhage, such as the FiO_2_, hematocrit, and platelet count. Moreover, it is noteworthy that most models unreasonably confuse late‐onset IVH with early‐onset, posing a high risk of bias. The present study was performed to construct a new prediction model for severe IVH. The data for this population‐based study came from a children's hospital. After screening by inclusion and exclusion criteria, 1009 very low birth weight infants (VLBWIs) were subsequently recruited in the study and divided into training and validation sets in a ratio of 7:3. Gestational age, Max FiO_2_, hematokrit on admission < 45%, and platelet count on admission < 100 × 10^9^/L were incorporated into the nomogram chart. The area under the curve (AUC) values demonstrated robust predictive performance, with the training set yielding an AUC of 0.884 (bootstrap‐corrected AUC = 0.903) and the validation set achieving an AUC of 0.859. The Delong test showed no statistically significant difference in AUCs between the training set and validation set (*p* = 0.528). The result of the Hosmer–Lemeshow test showed the model is well calibrated (*p* = 0.757). The present study identified the predictor model associated with severe IVH during the first 7 days of life, and the nomogram performed soundly, which would be a promising tool for early stratification of the risk for severe IVH in VLBWIs.

## 1 Introduction

Intraventricular hemorrhage (IVH) is the most common intracranial hemorrhage in premature infants, with approximately 50% occurring on the first day of life and about 90% within the first 3 days after birth [1]. The prognosis for premature infants worsens with increasing IVH severity, with mortality rates ranging from 18% to 40% for severe IVH [2]. Additionally, the probability of developing cerebral palsy in survivors of severe IVH is as high as 50% [3, 4]. The lower the gestational age and birth weight, the higher the likelihood of severe IVH. The incidence of severe IVH in very low birth weight infants (VLBWIs) is estimated to be between 7.0% and 13.1% [5, 6, 7, 8]. Given the lack of effective treatments for severe IVH, early prevention and identification are crucial.

The diagnosis of IVH primarily relies on bedside cranial ultrasound in neonatal intensive care units (NICUs). The American Academy of Pediatrics (AAP) recommends the first routine screening at around 7–10 days of age [9], whereas in countries like China, screening is advised within the first 3 days after birth [10]. Although some countries have adopted earlier screening protocols, factors such as varying admission ages, limited availability of professional ultrasound physicians, and insufficient equipment contribute to inconsistencies in ultrasound screening across different centers [11]. Nevertheless, timely screening for severe IVH remains essential.

In recent years, several risk prediction models for severe IVH in premature infants have been developed based on perinatal clinical data [12, 13, 14, 15, 16, 17, 18, 19, 20]. The most commonly used predictors include gestational age, sex, antenatal corticosteroid treatment, Apgar score at 1 min, birth weight, and delivery mode. However, a systematic review on this topic noted that most published models do not account for the clinical course and hemostatic profile of newborns, highlighting the need for improved risk prediction and stratification [21]. Furthermore, risk factors for late‐onset IVH differ from those for early‐onset IVH, making it difficult to isolate the influence of clinical or patient factors beyond the perinatal period. Previous models have rarely distinguished between IVH onset times, which compromises their predictive accuracy [12, 13, 14, 15, 16, 17, 18, 19, 20].

Additionally, most models have been developed using premature infant cohorts from developed countries. Variations in IVH incidence among different cohorts are influenced by gestational age distribution and disparities in treatment and care across regions. These differences affect the predictive performance of existing models [22], making them unsuitable for direct application in other populations. Therefore, this study aims to establish and validate a risk prediction model for early severe IVH based on the Chinese premature infant population.

## 2 Patients and Methods

### 2.1 Study Design and Setting

We conducted a retrospective study. All included infants represented postnatal transfers from regional maternity centers, with standardized referral documentation enabling systematic capture of antenatal and early postnatal variables at the time of transfer. The transfer documentation protocol maintained data collection of birth center parameters, including detailed delivery records and initial stabilization measures, allowing comprehensive analysis of pre‐transfer predictors. The Strengthening the Reporting of Observational Studies in Epidemiology (STROBE) guidelines and the Transparent Reporting of a Multivariable Prediction Model for Individual Prognosis or Diagnosis (TRIPOD) guidelines were followed to standardize the reporting of this predictive model study [23, 24].

### 2.2 Inclusion and Exclusion Criteria

Neonates hospitalized in the Level III NICU between June 2017 and December 2023 were screened for inclusion in this study.

Inclusion criteria: VLBWIs with a gestational age of < 37 weeks who were admitted to the NICU of the children's hospital within 24 h after birth.

Exclusion criteria: (1) Neonates with genetic metabolic diseases or life‐threatening congenital deformities. (2) Neonates who did not undergo cranial ultrasound within the first seven days after birth.

### 2.3 Sample Size Estimation

The sample size was calculated using R software (version 4.2.1) based on clinical prediction model guidelines [25]. The incidence of severe IVH in VLBWIs ranges from 7.0% to 13.1% [5, 6, 7, 8], with the present study adopting the higher value of 13.1%. The assumed incidence rate for each predicted parameter (events per predictor parameter) is 0.13. According to a previous study [15], the *R* ^2^ value is 0.44. Assuming an acceptable difference of 0.05 between the model's apparent and adjusted *R* ^2^, an intercept estimation error of 0.05, and 10 predictive factors, the minimum required sample size for model development is 276 cases. Considering an estimated dropout rate of 10%, at least 304 subjects should be included.

### 2.4 Data Collection

Due to the complex etiology of neonatal IVH, risk factors before, during, and shortly after delivery were comprehensively considered by reviewing factors mentioned in previous literature and incorporating objective recommendations from systematic reviews [21]. All data were retrieved from the hospital information system (HIS) and included the following: (1) Maternal data: Maternal age, hypertension, diabetes, heart diseases, chorioamnionitis, pregnancy mode, number of fetuses, premature rupture of membranes, intrauterine distress, prenatal glucocorticoid use, mode of delivery, amniotic fluid status, and so forth. (2) Neonatal data: Gestational age, birth weight, sex, Apgar score, resuscitation, pulmonary surfactant administration, transportation, age at NICU admission, weight at NICU admission, diagnosis, respiratory support, umbilical vascular catheterization, medication, and laboratory tests. All data were collected by two independent researchers and verified by a third researcher.

### 2.5 Grading of IVH

This study primarily predicts severe IVH occurring within the first 7 days after birth. IVH is classified into four grades: Grade I: Bleeding limited to the germinal matrix; Grade II: blood volume occupying ≤ 50% of the lateral ventricle; Grade III: blood volume occupying > 50% of the lateral ventricle; Grade IV: Hemorrhagic cerebral infarction on the same side as the lateral ventricle [26, 27].

For this study, mild IVH includes Grades I and II, while severe IVH includes Grades III and IV. IVH was routinely screened by a professional ultrasound specialist within 72 h after birth using bedside cranial ultrasound (Vivid e80, GE, US). If signs of IVH were detected, cranial ultrasound was performed every 2 days for follow‐up. The final IVH diagnosis was based on the most severe grade observed within the first 7 days after birth.

### 2.6 Neonatal Resuscitation

Neonates received warmth and positive pressure ventilation via a mask, with respiratory support provided by a T‐piece after birth. The need for further endotracheal intubation was determined by the neonatologist in the delivery room. Intubation was considered essential if continuous positive pressure ventilation via T‐piece was required, if mask positive pressure ventilation was ineffective, or if extrathoracic cardiac compression was performed. After resuscitation, the newborn was transferred to the NICU for further treatment. Neonatal resuscitation was conducted following the Chinese Neonatal Resuscitation Guidelines [28].

### 2.7 Data Analysis and Model Development

Data analysis was performed using R software (version 4.2.1). Quantitative data following a normal distribution were expressed as mean ± standard deviation (SD), and comparisons between two groups were conducted using an independent sample *t* test. Non‐normally distributed data were represented by the median and interquartile range (IQR), with comparisons between groups performed using the Mann–Whitney *U* test. Qualitative data were expressed as *n* (%), and intergroup comparisons were conducted using the chi‐square test or Fisher's exact test.

The dataset was randomly divided into a training set (70%) and a testing set (30%). The least absolute shrinkage and selection operator (LASSO) regression model was applied to the training set to identify predictive factors. Selected predictors underwent backward multivariate logistic regression to determine independent risk factors, with model optimization guided by Akaike's Information Criterion (AIC) minimization. A nomogram was then developed using the identified variables.

The predictive model's performance and calibration were assessed by plotting the receiver operating characteristic (ROC) curve and the calibration curve (Bootstrap = 1000). The DeLong test was used to compare the area under the curve (AUC) between the training and validation sets, while the Hosmer–Lemeshow goodness‐of‐fit test evaluated model calibration. Decision curve analysis (DCA) was performed to assess the clinical utility of the model.

## 3 Results

### 3.1 Participant Characteristics

**FIGURE 1:**
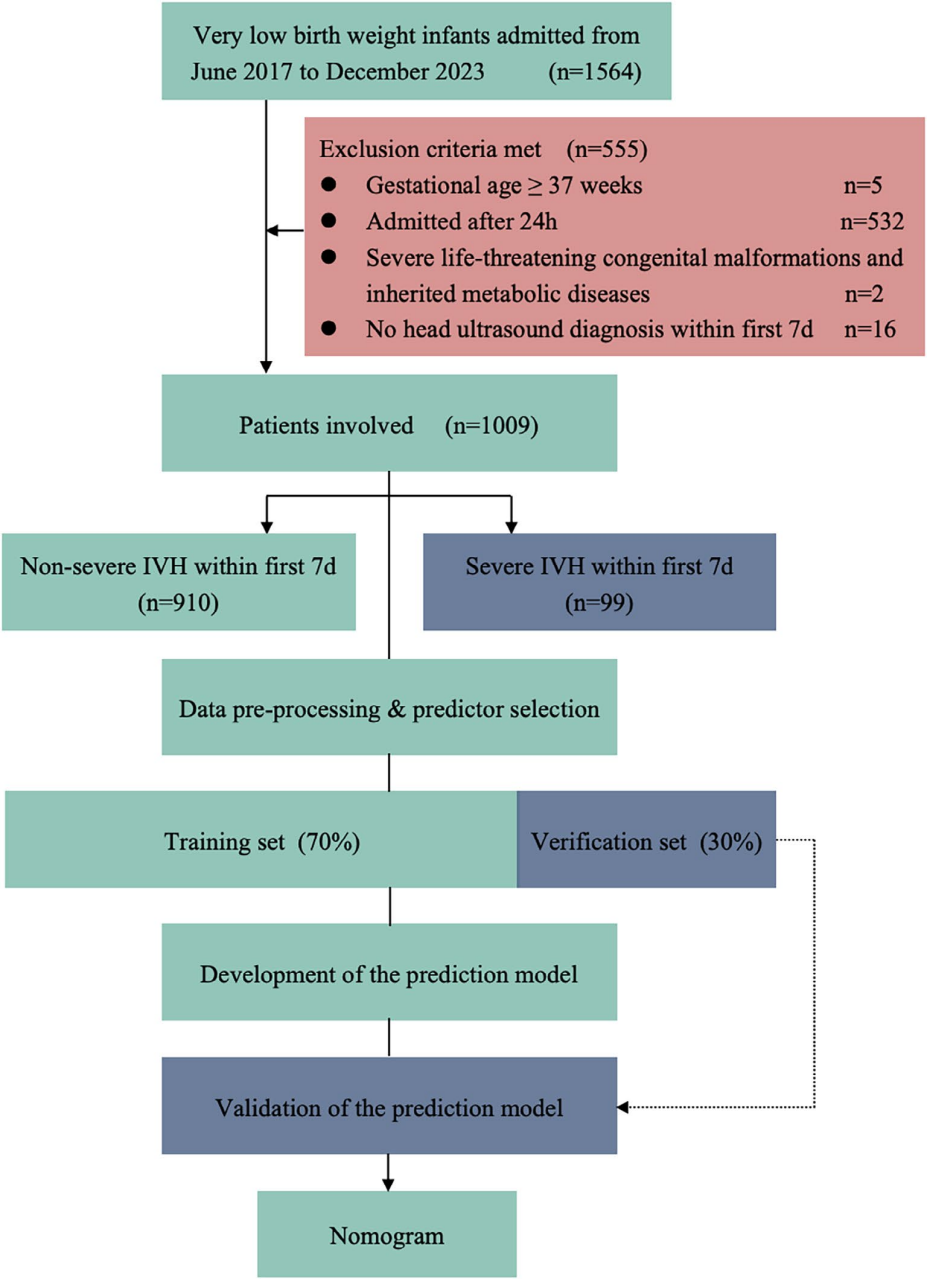
Flowchart of this study.

A total of 1009 VLBWIs met the inclusion and exclusion criteria (Figure 1). The cohort included 485 females (48.07%) and 524 males (51.93%), with a mean gestational age of 29.74 ± 2.35 weeks and a mean birth weight of 1172.81 ± 225.56 g. Approximately 9.81% of participants were diagnosed with severe IVH within 7 days of birth (Table 1).

**TABLE 1:** Baseline characteristics of the study population.

Variables	Total (*n* = 1009)	Non‐severe IVH (*n* = 910)	Severe IVH (*n* = 99)	Statistic	*p*
*Neonatal characteristics*					
Gestational age, week, mean (SD)	29.74 ± 2.35	29.99 ± 2.22	27.42 ± 2.21	10.945	< 0.001
Birth weight, g, mean (SD)	1172.81 ± 225.56	1193.96 ± 213.63	978.41 ± 240.14	9.414	< 0.001
Male, *n* (%))	524 (51.93)	466 (51.21)	58 (58.59)	1.662	0.197
1‐min Apgar score, median (IQR)	8 (7, 8)	8 (8, 8)	7 (4, 8)	−8.822	< 0.001
1‐min Apgar score ≤ 7, *n* (%)	276 (27.35)	212 (23.30)	64 (64.65)	74.758	< 0.001
5‐min Apgar score, median (IQR)	9 (8, 9)	9 (9, 9)	8 (7, 9)	−8.451	< 0.001
5‐min Apgar score ≤ 7, *n* (%)	108 (10.70)	74 (8.13)	34 (34.34)	61.468	< 0.001
DR resuscitation, *n* (%)	233 (23.09)	173 (19.01)	60 (60.61)	84.657	< 0.001
DR intubation, *n* (%)	107 (10.60)	70 (7.69)	37 (37.37)	79.874	< 0.001
DR chest compression, *n* (%)	28 (2.78)	18 (1.98)	10 (10.10)	Fisher	< 0.001
DR epinephrine, *n* (%)	27 (2.68)	19 (2.09)	8 (8.08)	Fisher	0.003
DR surfactant, *n* (%)	35 (3.47)	27 (2.97)	8 (8.08)	Fisher	0.016
Transport time after birth, h, median (IQR)	0.68 (0.68, 1.68)	0.68 (0.68, 1.68)	0.68 (0.68, 4.94)	−2.555	0.011
Transport duration, h, median (IQR)	0.16 (0.16, 0.16)	0.16 (0.16, 0.16)	0.16 (0.16, 0.33)	−4.042	< 0.001
Transport radius, km, median (IQR)	3 (3, 3)	3 (3, 3)	3 (3, 20)	−4.040	< 0.001
IMV during transportation, *n* (%)	304 (30.13)	229 (25.16)	75 (75.76)	106.173	< 0.001
Age on admission, h, median (IQR)	1 (1, 2)	1 (1, 2)	1 (1, 6.5)	−3.289	0.001
Weight on admission, g, mean (SD)	1163.69 ± 226.33	1185.63 ± 214.00	962.02 ± 237.79	9.762	< 0.001
RDS on admission, *n* (%)	749 (74.23)	652 (71.65)	97 (97.98)	31.002	< 0.001
NIV on admission, *n* (%)	412 (40.83)	396 (43.52)	16 (16.16)	26.534	< 0.001
IMV on admission, *n* (%)	351 (34.79)	270 (29.67)	81 (81.82)	104.744	< 0.001
Max PEEP, median (IQR)	6 (5, 6)	6 (5, 6)	6 (6, 6)	−4.516	< 0.001
Max FiO_2_, median (IQR)	30 (25, 38)	30 (25, 35)	50 (30, 75)	−9.920	< 0.001
Max FiO_2_ > 30%, *n* (%)	687 (68.09)	594 (65.27)	93 (93.94)	32.457	< 0.001
Max FiO_2_ > 40%, *n* (%)	252 (24.98)	188 (20.66)	64 (64.65)	89.866	< 0.001
Max FiO_2_ > 60%, *n* (%)	90 (8.92)	53 (5.82)	37 (37.37)	105.546	< 0.001
Lowest SpO_2_ on admission < 90%, *n* (%)	209 (20.71)	173 (19.01)	36 (36.36)	15.331	< 0.001
Lowest SpO_2_ on admission < 85%, *n* (%)	77 (7.63)	61 (6.70)	16 (16.16)	10.029	0.002
Body temperature on admission < 36.0°C, *n* (%)	502 (49.75)	456 (50.11)	46 (46.46)	0.34	0.56
Body temperature on admission < 36.5°C, *n* (%)	877 (86.92)	797 (87.58)	80 (80.81)	3.032	0.082
Hypotension on admission, *n* (%)	165 (16.35)	139 (15.27)	26 (26.26)	7.098	0.008
Blood glucose on admission < 2.6 mmol/L, *n* (%)	134 (13.28)	120 (13.19)	14 (14.14)	0.012	0.913
Blood glucose on admission < 2.2 mmol/L, *n* (%)	92 (9.12)	80 (8.79)	12 (12.12)	0.827	0.363
Blood glucose on admission > 7.0 mmol/L, *n* (%)	91 (9.02)	70 (7.69)	21 (21.21)	18.276	< 0.001
Dopamine hydrochloride, *n* (%)	336 (33.30)	276 (30.33)	60 (60.61)	35.498	< 0.001
Dobutamine hydrochloride, *n* (%)	451 (44.70)	384 (42.20)	67 (67.68)	22.429	< 0.001
NICU surfactant, *n* (%)	516 (51.14)	435 (47.80)	81 (81.82)	39.996	< 0.001
NICU transfusion, *n* (%)	96 (9.51)	69 (7.58)	27 (26.27)	37.955	< 0.001
Umbilical vein catheterization, *n* (%)	372 (36.87)	322 (35.38)	50 (50.51)	8.133	0.004
NICU epinephrine, *n* (%)	38 (3.77)	21 (2.31)	17 (16.17)	Fisher	< 0.001
NICU caffeine, *n* (%)	641 (63.53)	570 (62.64)	71 (71.72)	2.797	0.094
RBC on admission < 4.4 × 10^12^/L, *n* (%)	590 (58.47)	506 (55.60)	84 (84.85)	30.254	< 0.001
Hemoglobin on admission < 114 g/L, *n* (%)	38 (3.77)	26 (2.86)	12 (12.12)	Fisher	< 0.001
Hemoglobin on admission < 100 g/L, *n* (%)	18 (1.78)	12 (1.32)	6 (6.06)	Fisher	0.005
Hematokrit on admission < 28%, *n* (%)	15 (1.49)	9 (0.99)	6 (6.06)	Fisher	0.002
Hematokrit on admission < 45%, *n* (%)	241 (23.89)	188 (20.66)	53 (53.54)	51.289	< 0.001
Platelet count on admission < 100 × 10^9^/L, *n* (%)	76 (7.53)	55 (6.04)	21 (21.21)	27.357	< 0.001
Platelet count on admission < 150 × 10^9^/L, *n* (%)	239 (23.69)	200 (21.98)	39 (39.39)	14.034	< 0.001
Total protein on admission < 44 g/L, *n* (%)	626 (62.04)	544 (59.78)	82 (82.83)	19.173	< 0.001
Albumin on admission < 32.8 g/L, *n* (%)	733 (72.65)	641 (70.44)	92 (92.93)	21.608	< 0.001
Globulin on admission < 8.8 g/L, *n* (%)	218 (21.61)	192 (21.10)	26 (26.26)	1.117	0.291
K^+^ < 4.6 mmol/L, *n* (%)	236 (23.39)	217 (23.85)	19 (19.19)	0.835	0.361
K^+^ > 6.7 mmol/L, *n* (%)	57 (5.65)	53 (5.82)	4 (4.04)	0.251	0.616
Na^+^ < 133 mmol/L, *n* (%)	35 (3.47)	27 (2.97)	8 (8.08)	Fisher	0.016
Na^+^ > 146 mmol/L, *n* (%)	44 (4.36)	33 (3.63)	11 (11.11)	Fisher	0.002
Ca^2+^ < 1.53 mmol/L, *n* (%)	24 (2.38)	17 (1.87)	7 (7.07)	Fisher	0.006
*Maternal characteristics*					
Maternal age, y, mean (SD)	30.79 ± 4.87	30.77 ± 4.91	30.93 ± 4.44	−0.304	0.761
PIH, *n* (%)	201 (19.92)	188 (20.66)	13 (13.13)	2.718	0.099
Maternal diabetes, *n* (%)	157 (15.56)	141 (15.49)	16 (16.16)	0.001	0.978
Maternal heart diseases, *n* (%)	21 (2.08)	15 (1.65)	6 (6.06)	Fisher	0.012
Clinical chorioamnionitis, *n* (%)	25 (2.48)	17 (1.87)	8 (8.08)	11.807	0.001
Test tube baby, *n* (%)	235 (23.29)	196 (21.54)	39 (39.39)	14.949	< 0.001
Multiple births, *n* (%)	378 (37.46)	332 (36.48)	46 (46.46)	3.383	0.066
Placental abruption, *n* (%)	31 (3.07)	26 (2.86)	5 (5.05)	Fisher	0.219
Premature rupture of membranes ≥ 24 h, *n* (%)	279 (27.65)	250 (27.47)	29 (29.29)	0.071	0.79
Fetal distress, *n* (%)	71 (7.04)	64 (7.03)	7 (7.07)	0	1
Antenatal steroids complete, *n* (%)	328 (32.51)	304 (33.41)	24 (24.24)	3.013	0.083
Antenatal steroids partial, *n* (%)	318 (31.52)	297 (32.64)	21 (21.21)	4.884	0.027
Vaginal delivery, *n* (%)	422 (41.82)	358 (39.34)	64 (64.65)	22.471	< 0.001
Clear amniotic fluid, *n* (%)	919 (91.08)	842 (92.53)	77 (77.78)	22.129	< 0.001
Normal amniotic fluid volume, *n* (%)	934 (92.57)	846 (92.97)	88 (88.89)	1.606	0.205

Abbreviations: DR, delivery room; FiO_2_, fraction of inspired oxygen; IMV, invasive mechanical ventilation; IQR, interquartile range; NICU, neonatal intensive care unit; NIV, noninvasive ventilation; PEEP, positive end expiratory pressure; PIH, pregnancy‐induced hypertension; RBC, red blood cell count; RDS, respiratory distress syndrome; SD, standard deviation; SpO_2_, pulse oxygen saturation.

All patients were randomly assigned to either the training group (*n* = 706) or the validation group (*n* = 303). The baseline characteristics of both groups were comparable, with no statistically significant differences (*p* > 0.05), as shown in Table S1. A comparison of positive and negative cases within the training set is provided in Table S2.

### 3.2 LASSO Regression and Logistic Regression Analysis

LASSO regression was performed for variable selection, using 10‐fold cross‐validation to determine the optimal Lambda (λ) value. The one‐standard‐error rule (λ 1‐SE) was applied, resulting in the selection of seven predictive factors.

**FIGURE 2:**
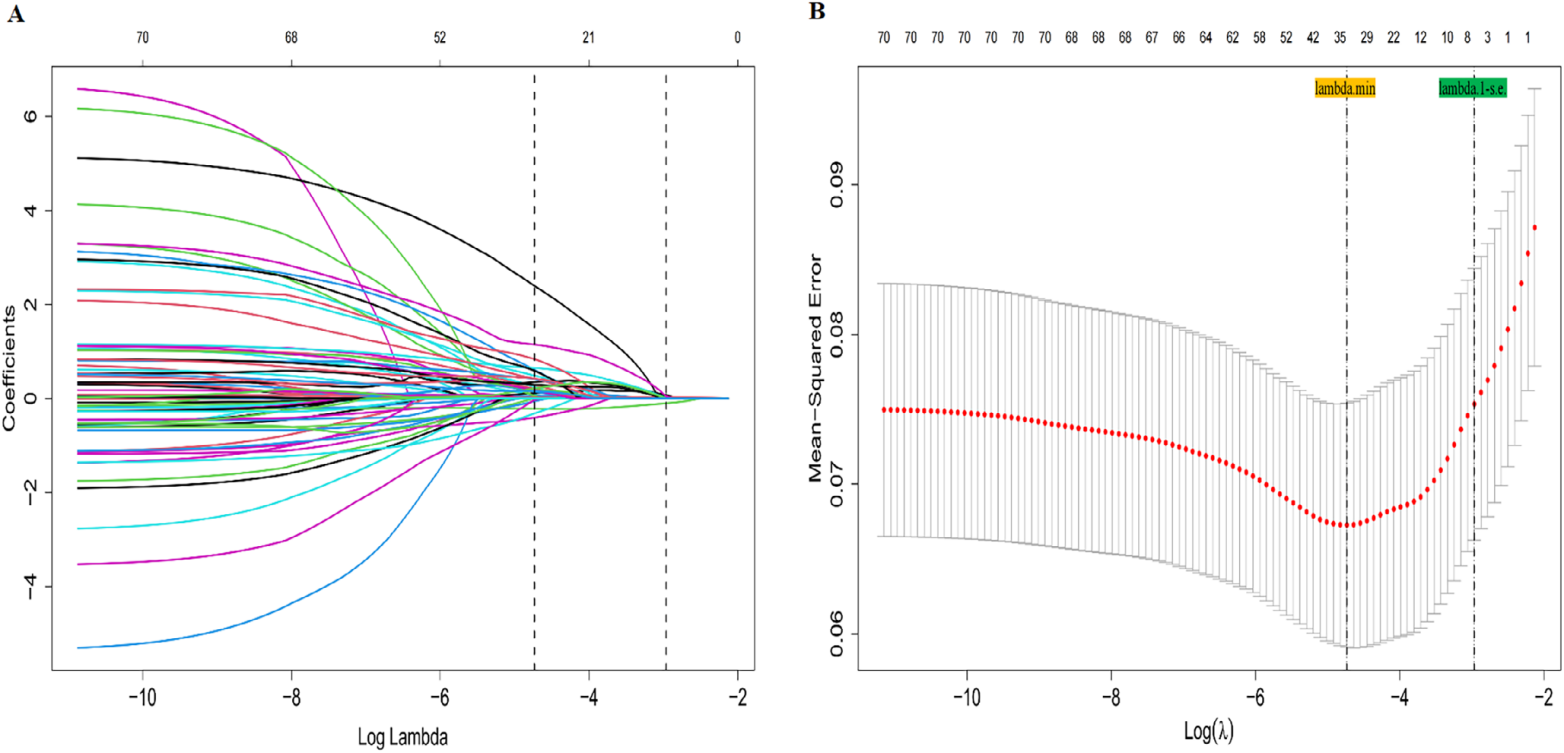
Graph of LASSO regression. (A) The horizontal axis represents the Log Lambda value and the vertical axis represents the coefficient. (B) The horizontal axis represents the Log Lambda value and the vertical axis represents mean squared error.

Multivariate logistic regression analysis was then conducted using these factors. The results indicated that delivery room resuscitation, weight on admission, and invasive mechanical ventilation (IMV) were not significantly associated with severe IVH (*p* > 0.05). Ultimately, gestational age, maximum FiO_2_, HCT < 45% at admission, and platelet count < 100 × 10^9^/L at admission were identified as independent predictors of severe IVH (Table 2, Figure 2).

**TABLE 2:** Predictive factors screened by multivariate logistic regression.

Characteristics	*B*	SE	OR (95% CI)	*Z*	*p*
(Intercept)	11.015	2.53681	60763.762 (490.5–10,597)	4.342	0
Gestational age	−0.526	0.08824	0.591 (0.493–0.697)	−5.958	0
Max FiO_2_	0.029	0.00643	1.029 (1.016–1.042)	4.496	0
Hematokrit on admission < 45%	0.863	0.31531	2.369 (1.269–4.389)	2.735	0.006
Platelet count on admission < 100 × 10^9^/L	2.073	0.43761	7.949 (3.360–18.87)	4.737	0

Abbreviations: CI, confidence interval; FiO_2_, fraction of inspired oxygen.

### 3.3 Nomogram Development

**FIGURE 3:**
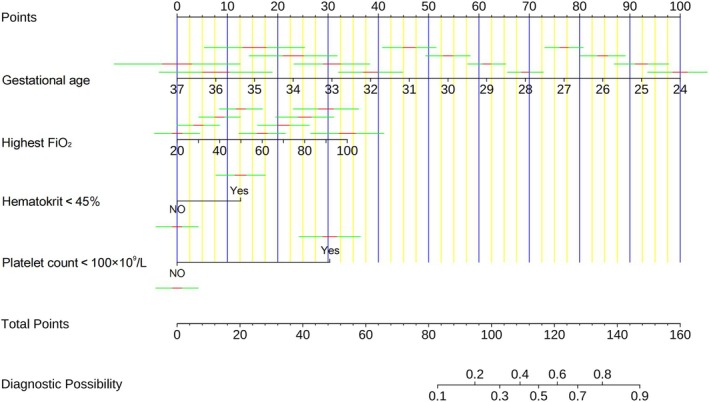
Nomogram chart based on multivariate logistic regression.

A predictive model incorporating four variables—gestational age, max FiO_2_, HCT < 45% at admission, and platelet count < 100 × 10^9^/L at admission—was developed based on LASSO and logistic regression analyses. The model was visualized as a nomogram (Figure 3).

For example, if a premature infant is admitted within 24 h of birth with a gestational age of 28 weeks, a required max FiO_2_ of 40%, HCT > 45% at admission, and a platelet count < 100 × 10^9^/L, the total score in the nomogram is 110 points. The corresponding probability of severe IVH, as indicated in the nomogram, is approximately 0.4.

### 3.4 Model Performance Evaluation

**FIGURE 4:**
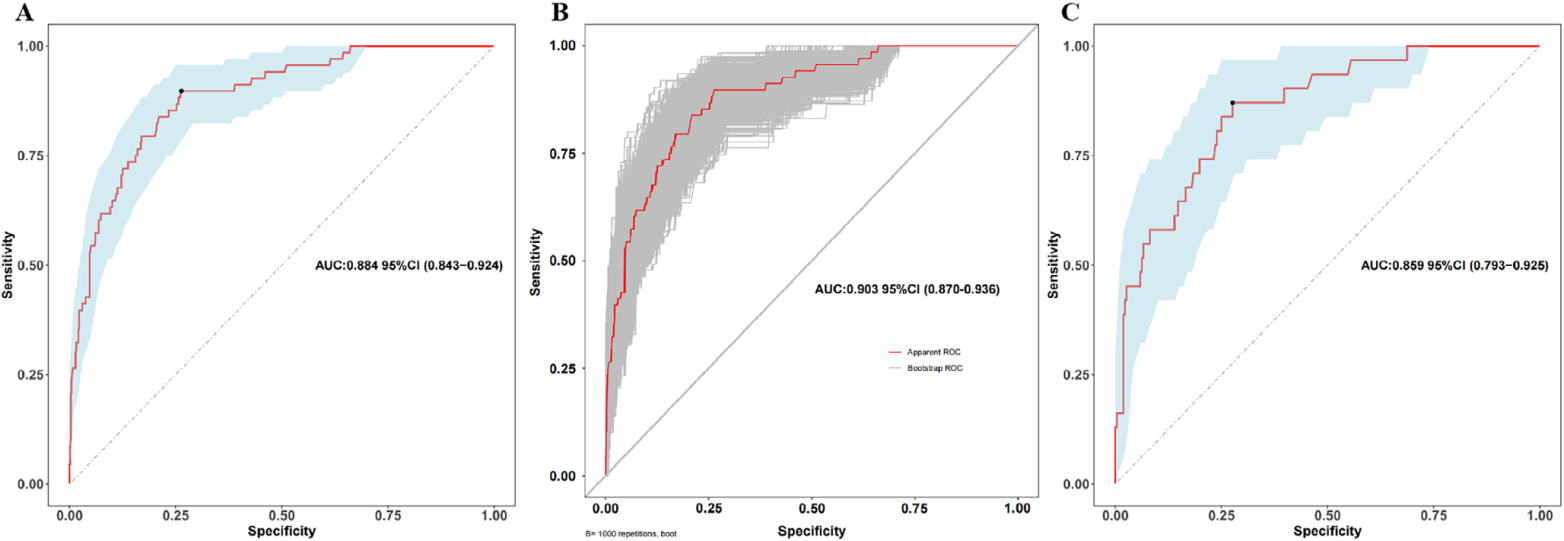
ROC of prediction risk model. (A) Training set; (B) the bias‐corrected outcome for the training set following bootstrap adjustment with 1000 iterations; (C) test set.

The AUC for the training set was 0.884 (95% CI: 0.843–0.924), with a bootstrap‐corrected AUC of 0.903 (95% CI: 0.870–0.936), indicating strong discriminatory ability. In the validation set, the AUC was 0.859 (95% CI: 0.793–0.925), further confirming the model's robustness (Figure 4). The DeLong test showed no statistically significant difference in AUCs between the training and validation sets (*D* = 0.63104, df = 539.31, *p* = 0.5283).

**FIGURE 5:**
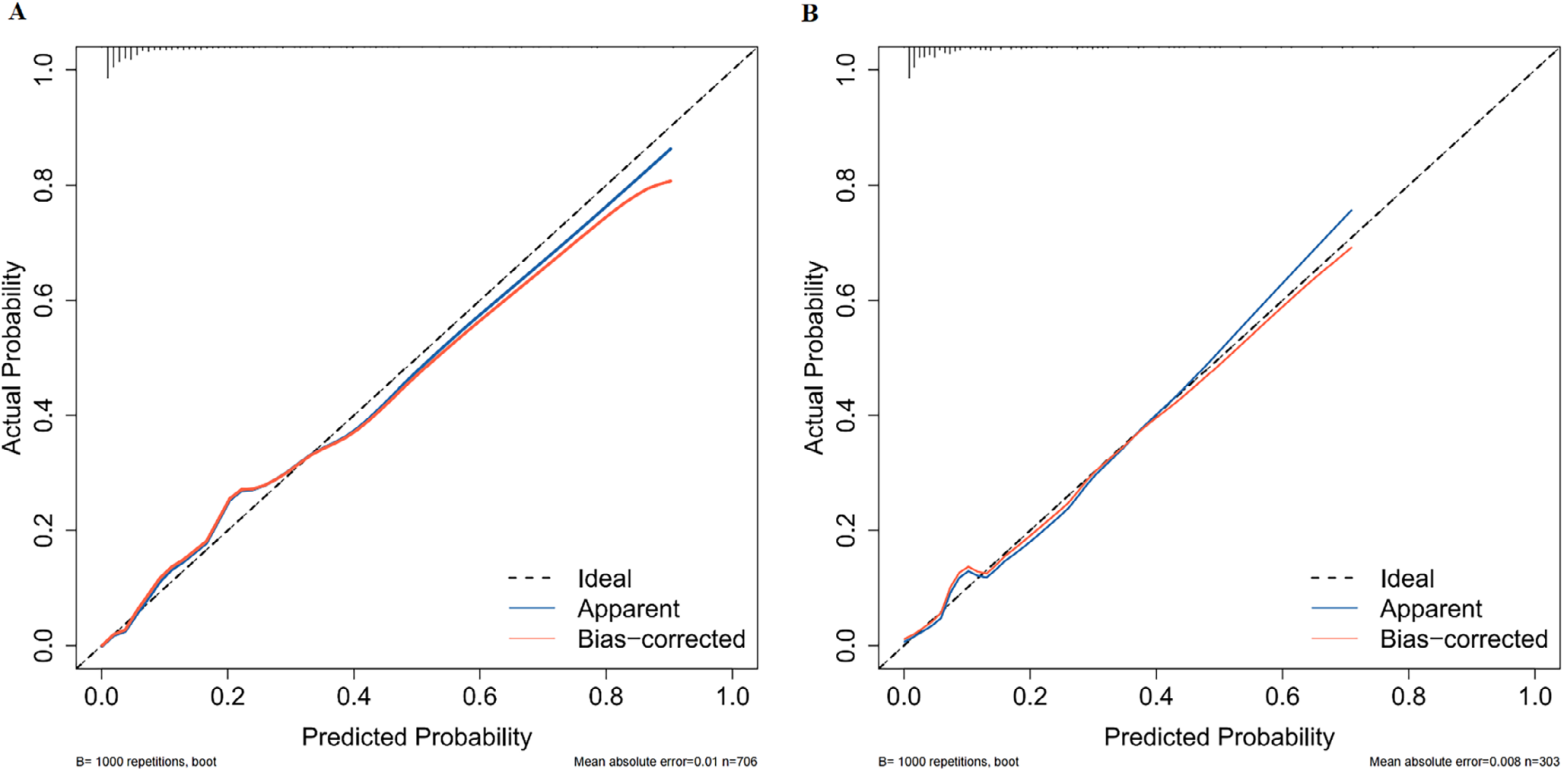
Calibration curve of prediction risk model. The blue line represents the entire cohort, and the red line is the result after bias correction by bootstrapping (1000 repetitions). (A) Training set; (B) test set.

The Brier score for the training set was 0.062, with a calibration intercept of 0 and a calibration slope of 1. The Hosmer–Lemeshow test yielded *X* ^2^ = 4.2602, df = 8, *p* = 0.8329, indicating good model calibration. In the validation set, the Brier score was 0.068, the calibration intercept was 0.176, and the calibration slope was 1.017. The Hosmer‐Lemeshow test showed *X* ^2^ = 5.01, df = 8, *p* = 0.7565 (Figure 5).

**FIGURE 6:**
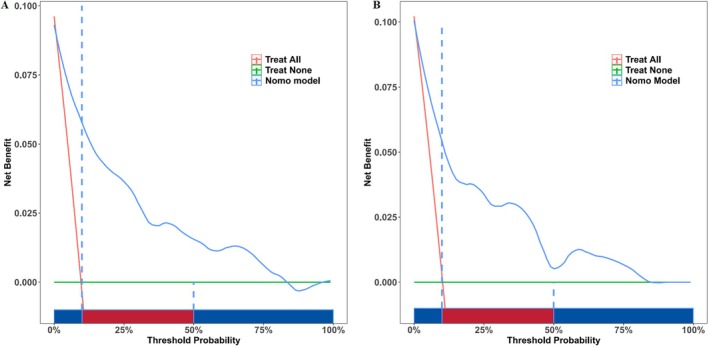
DCA of prediction risk model. (A) Training set; (B) test set.

DCA demonstrated that when the threshold probability for predicting severe IVH ranged from 10% to 90%, the model provided a net benefit in both the training and validation sets (Figure 6).

## 4 Discussion

This study established the first simple and quantifiable nomogram by effectively screening clinical features before, during, and early postpartum delivery to predict the risk of severe IVH in VLBWIs within 7 days of birth. Premature birth is the leading cause of death in children under 5 years old, and severe IVH is a major contributor to mortality in preterm infants [29]. This model offers a promising tool for early risk stratification of severe IVH, aiding in the early clinical prevention of its occurrence and progression.

To the best of our knowledge, only He et al. [30] have developed a scoring system for severe IVH using the Chinese VLBWI cohort. However, their model relied on variables recorded within 5 days of birth, which do not align with the objective of predicting and identifying severe IVH as early as possible. Our model is tailored to the conditions of developing countries, as all predictive variables can be obtained within 24 h of birth without requiring complex diagnostic equipment.

Multivariate logistic regression analysis identified gestational age as a protective factor against severe IVH, while high FiO_2_ concentration, HCT > 45% at admission, and platelet count < 100 × 10^9^/L at admission were independent risk factors. Gestational age plays a crucial role in predictive models due to the inherent fragility of the germinal matrix microvasculature and immature cerebral blood flow autoregulation [31]. This variable is consistently included in most published predictive models [12, 13, 15, 16, 17, 19, 20]. A national multicenter study in China confirmed that lower gestational age is associated with a higher incidence of severe IVH, with the risk increasing by approximately 25% per week of gestational age reduction [8]. However, due to the complex pathophysiology of IVH—including immature vascular anatomy and hemodynamic instability—gestational age alone is insufficient for predicting adverse outcomes [19, 32]. Despite clinical interventions such as maintaining a midline head position, tilting the incubator 15°–30°, avoiding head‐down positioning and sudden leg elevation, and preventing rapid intravenous/arterial flushes or withdrawals [33], severe IVH remains a significant risk in very preterm infants.

Another key finding is that higher FiO_2_ at admission is an independent risk factor for severe IVH. This aligns with Fairchild et al. [34] who demonstrated the predictive value of the Respiratory Acuity Score (calculated using FiO_2_ levels) for severe IVH. Similarly, Linder et al. [14] found that infants with severe IVH required higher FiO_2_ concentrations to maintain target oxygenation within the first 24 h of birth. Luque et al. [17] included respiratory distress syndrome and mechanical ventilation as predictive variables, further suggesting an association between severe respiratory disease and IVH. A decrease in oxygen supply may trigger an increase in cerebral blood flow to maintain normal SaO_2_ levels [35]. A systematic review found that lower SpO_2_ at 5 min post‐birth is associated with an increased risk of adverse outcomes, including severe IVH [36]. Therefore, when low‐gestational‐age preterm infants require high‐concentration oxygen therapy at admission, clinicians should be vigilant for potential severe IVH.

This study also found that lower hematocrit and platelet counts within 24 h of birth were associated with a higher incidence of IVH. Weinstein et al. [37] and Dekom et al. [38] demonstrated that HCT < 45% correlates with an increased risk of IVH, with the latter study reporting a twofold increase in IVH incidence. Notably, our study is the first to confirm the predictive value of HCT < 45% using a multivariate model. Low hematocrit can affect blood viscosity, cerebral blood flow perfusion, and hemodynamic stability, increasing the risk of bleeding. Thrombocytopenia has also been strongly linked to IVH. A multicenter cohort study in the United States confirmed its association with increased IVH risk [39], while a recent Chinese study identified a correlation between platelet count and IVH of any grade [40]. In our model, a platelet count < 100 × 10^9^/L was closely associated with severe IVH, reinforcing the importance of hematologic parameters in early risk assessment.

## 5 Clinical Implications

Currently, there is a lack of convenient and effective predictive models in clinical practice, particularly in developing countries. The predictive indicators included in this model are easily obtainable in the early postnatal period, enhancing its practicality. The DCA results indicate high clinical applicability, supporting its use in managing premature infants. This model can help clinicians allocate limited resources more effectively by identifying high‐risk populations and implementing targeted preventive measures.

## 6 Limitations

Despite strong internal validation in terms of discriminability, calibration, and clinical applicability, this study has several limitations. First, as a retrospective study conducted in a standalone children's hospital where all neonates were postnatal transfers, our electronic medical records lack routine documentation of maternal antenatal MgSO_4_ administration from referring institutions. This may limit the assessment of fetal neuroprotective interventions. However, MgSO_4_ is rarely retained as an independent predictor in contemporary IVH risk models, likely due to its widespread use in modern perinatal care, which diminishes its discriminative value.

Second, the data span 7 years, during which updates in premature infant management may have influenced outcomes. Finally, we cannot completely rule out the possibility that some cases of IVH may have already occurred in utero, introducing a potential reverse causal relationship between predictive variables and outcomes. However, intrauterine IVH accounts for only a small proportion of cases, making it unlikely to affect the overall conclusions.

Future research should focus on prospective studies and external validation across diverse ethnic populations, incorporating standardized obstetric‐neonatal data linkages and additional predictive variables (e.g., blood gas analysis). This iterative approach will allow continuous model refinement through the integration of emerging biomarkers, advancements in perinatal care, and evaluation of population‐specific risk modifiers.

## 7 Conclusion

This study identified four key predictive variables (gestational age, max FiO_2_ upon admission, hematocrit, and platelet count) and developed a visual nomogram to simplify complex regression equations. This model provides a practical risk stratification tool for early prediction of severe IVH within 7 days of birth, facilitating more accurate diagnosis and targeted prevention in clinical practice.

## Ethics Statement

This study was approved by the Ethics Committee of the Children's Hospital of Nanjing Medical University (No.: 202401020‐1). All data were fully anonymized before further statistical analysis. All the procedures were followed in accordance with the Declaration of Helsinki.

## Conflicts of Interest

The authors declare no conflicts of interest.

## Supporting information


**Table S1.** Baseline characteristics between training set and testing set.
**Table** **S2**. Comparison of positive and negative data in the training set.

## Data Availability

The data that support the findings of this study are available from the corresponding author upon reasonable request.
