# Supplementary material for: Construction and Validation of a Risk Prediction Model for Early Severe Intraventricular Hemorrhage in Very Low Birth Weight Infants
Source: Kaohsiung J Med Sci. 2025 May 19;41(7):e70037. doi: 10.1002/kjm2.70037 (PMC12412569; doi:10.1002/kjm2.70037)
Supplement: Supplementary file 1 — Table S1. Baseline characteristics between training set and testing set. Table S2 . Comparison of positive and negative data in the training set. [file KJM2-41-e70037-s001.docx]

Supplementary-Table 1. Baseline characteristics between training set and testing set

| **Variables** | **Total (n = 1009)** | **Test (n = 303)** | **Train (n = 706)** | **statistic** | ***p-value*** |
| --- | --- | --- | --- | --- | --- |
| **Neonatal characteristics** |  |  |  |  |  |
| Gestational age, wk, mean (SD) | 29.74±2.35 | 29.69±2.35 | 29.76±2.35 | 0.415 | 0.678 |
| Birth weight, g, mean (SD) | 1172.81±225.56 | 1169.40±236.60 | 1174.27±220.80 | 0.315 | 0.753 |
| Male, n (%) | 524 (52) | 150 (50) | 374 (53) | 0.888 | 0.346 |
| 1-min Apgar score, Median (IQR) | 8 (7, 8) | 8 (7, 8) | 8 (7, 8) | -0.605 | 0.545 |
| 1-min Apgar score≤7, n (%) | 276 (27) | 78 (26) | 198 (28) | 0.456 | 0.5 |
| 5-min Apgar score, Median (IQR) | 9 (8, 9) | 9 (8, 9) | 9 (8, 9) | -0.262 | 0.793 |
| 5-min Apgar score≤7, n (%) | 108 (11) | 32 (11) | 76 (11) | 0 | 1 |
| DR resuscitation, n (%) | 233 (23) | 63 (21) | 170 (24) | 1.112 | 0.292 |
| DR intubation, n (%) | 107 (11) | 28 (9) | 79 (11) | 0.656 | 0.418 |
| DR chest compression, n (%) | 28 (3) | 6 (2) | 22 (3) | 0.637 | 0.425 |
| DR epinephrine, n (%) | 27 (3) | 8 (3) | 19 (3) | 0 | 1 |
| DR surfactant, n (%) | 35 (3) | 8 (3) | 27 (4) | 0.569 | 0.451 |
| Transport time after birth, h, Median (IQR) | 0.68 (0.68, 1.68) | 0.68 (0.68, 0.68) | 0.68 (0.68, 1.68) | -1.608 | 0.108 |
| Transport duration, h, Median (IQR) | 0.16 (0.16, 0.16) | 0.16 (0.16, 0.16) | 0.16 (0.16, 0.16) | -0.061 | 0.951 |
| Transport radius, km, Median (IQR) | 3 (3, 3) | 3 (3, 3) | 3 (3, 3) | -0.090 | 0.928 |
| IMV during transportation, n (%) | 304 (30) | 81 (27) | 223 (32) | 2.148 | 0.143 |
| Age on admission, h, Median (IQR) | 1 (1, 2) | 1 (1, 2) | 1 (1, 2) | -1.780 | 0.075 |
| Weight on admission, g, mean (SD) | 1163.69±226.33 | 1161.29±236.97 | 1164.72±221.77 | 0.221 | 0.825 |
| RDS on admission, n (%) | 749 (74) | 227 (75) | 522 (74) | 0.061 | 0.804 |
| NIV on admission, n (%) | 412 (41) | 136 (45) | 276 (39) | 2.708 | 0.1 |
| IMV on admission, n (%) | 351 (35) | 99 (33) | 252 (36) | 0.725 | 0.395 |
| Max PEEP, Median (IQR) | 6 (5, 6) | 6 (5.5, 6) | 6 (5, 6) | -1.235 | 0.217 |
| Max FiO_2_, Median (IQR) | 30 (25, 38) | 30 (25, 35) | 30 (25, 40) | -1.077 | 0.282 |
| Max FiO_2_＞30%, n (%) | 687 (68) | 203 (67) | 484 (69) | 0.171 | 0.679 |
| Max FiO_2_＞40%, n (%) | 252 (25) | 66 (22) | 186 (26) | 2.119 | 0.145 |
| Max FiO_2_＞60%, n (%) | 90 (9) | 26 (9) | 64 (9) | 0.016 | 0.899 |
| Lowest SpO_2_ on admission＜90%, n (%) | 209 (21) | 63 (21) | 146 (21) | 0 | 1 |
| Lowest SpO_2_ on admission＜85%, n (%) | 77 (8) | 23 (8) | 54 (8) | 0 | 1 |
| Body temperature on admission＜36.5℃, n (%) | 502 (50) | 262 (86) | 615 (87) | 0.031 | 0.861 |
| Body temperature on admission＜36.0℃, n (%) | 877 (87) | 139 (46) | 363 (51) | 2.388 | 0.122 |
| Hypotension on admission, n (%) | 165 (16) | 50 (17) | 115 (16) | 0 | 1 |
| Blood glucose on admission＜2.6mmol/L, n (%) | 134 (13) | 35 (12) | 99 (14) | 0.92 | 0.337 |
| Blood glucose on admission＜2.2mmol/L, n (%) | 92 (9) | 25 (8) | 67 (9) | 0.258 | 0.612 |
| Blood glucose on admission＞7.0mmol/L, n (%) | 91 (9) | 31 (10) | 60 (8) | 0.579 | 0.447 |
| Dopamine hydrochloride, n (%) | 336 (33) | 97 (32) | 239 (34) | 0.245 | 0.62 |
| Dobutamine hydrochloride, n (%) | 451 (45) | 136 (45) | 315 (45) | 0 | 0.993 |
| NICU surfactant, n (%) | 516 (51) | 160 (53) | 356 (50) | 0.39 | 0.532 |
| NICU transfusion, n (%) | 96 (10) | 29 (10) | 67 (9) | 0 | 1 |
| Umbilical vein catheterization, n (%) | 372 (37) | 113 (37) | 259 (37) | 0.013 | 0.911 |
| NICU epinephrine, n (%) | 38 (4) | 12 (4) | 26 (4) | 0.001 | 0.974 |
| NICU caffeine, n (%) | 641 (64) | 195 (64) | 446 (63) | 0.082 | 0.774 |
| RBC on admission＜4.4×10^12^/L, n (%) | 590 (58) | 186 (61) | 404 (57) | 1.346 | 0.246 |
| Hemoglobin on admission＜114g/L, n (%) | 38 (4) | 9 (3) | 29 (4) | 0.475 | 0.491 |
| Hemoglobin on admission＜100g/L, n (%) | 18 (2) | 4 (1) | 14 (2) | 0.221 | 0.639 |
| Hematokrit on admission＜28%, n (%) | 15 (1) | 3 (1) | 12 (2) | Fisher | 0.572 |
| Hematokrit on admission＜45%, n (%) | 241 (24) | 82 (27) | 159 (23) | 2.162 | 0.141 |
| Platelet count on admission＜100×10^9^/L, n (%) | 76 (8) | 18 (6) | 58 (8) | 1.265 | 0.261 |
| Platelet count on admission＜150×10^9^/L, n (%) | 239 (24) | 70 (23) | 169 (24) | 0.042 | 0.837 |
| Total protein on admission＜44g/L, n (%) | 626 (62) | 183 (60) | 443 (63) | 0.403 | 0.526 |
| Albumin on admission＜32.8g/L, n (%) | 733 (73) | 219 (72) | 514 (73) | 0.009 | 0.924 |
| Globulin on admission＜8.8g/L, n (%) | 218 (22) | 58 (19) | 160 (23) | 1.351 | 0.245 |
| K^+^＜4.6mmol/L, n (%) | 236 (23) | 72 (24) | 164 (23) | 0.01 | 0.919 |
| K^+^＞6.7mmol/L, n (%) | 57 (6) | 21 (7) | 36 (5) | 1.013 | 0.314 |
| Na^+^＜133mmol/L, n (%) | 35 (3) | 11 (4) | 24 (3) | 0 | 1 |
| Na^+^＞146mmol/L, n (%) | 44 (4) | 17 (6) | 27 (4) | 1.222 | 0.269 |
| Ca^2+^＜1.53mmol/L, n (%) | 24 (2) | 7 (2) | 17 (2) | 0 | 1 |
| **Maternal characteristics** |  |  |  |  |  |
| Maternal age, y, mean (SD) | 30.79±4.87 | 30.76±4.84 | 30.80±4.88 | 0.123 | 0.902 |
| PIH, n (%) | 201 (20) | 54 (18) | 147 (21) | 1.015 | 0.314 |
| Maternal diabetes, n (%) | 157 (16) | 50 (17) | 107 (15) | 0.199 | 0.656 |
| Maternal heart diseases, n (%) | 21 (2) | 8 (3) | 13 (2) | 0.33 | 0.566 |
| Clinical chorioamnionitis, n (%) | 25 (2.5) | 8 (2.6) | 17 (2.4) | 0.047 | 0.828 |
| Test tube baby, n (%) | 235 (23) | 70 (23) | 165 (23) | 0 | 0.991 |
| Multiple births, n (%) | 378 (37) | 121 (40) | 257 (36) | 0.983 | 0.321 |
| Placental abruption, n (%) | 31 (3) | 7 (2) | 24 (3) | 0.518 | 0.472 |
| Premature rupture of membranes≥24h, n (%) | 279 (28) | 91 (30) | 188 (27) | 1.064 | 0.302 |
| Fetal distress, n (%) | 71 (7) | 26 (9) | 45 (6) | 1.259 | 0.262 |
| Antenatal steroids complete, n (%) | 328 (33) | 96 (32) | 232 (33) | 0.086 | 0.77 |
| Antenatal steroids partial, n (%) | 318 (32) | 100 (33) | 218 (31) | 0.351 | 0.554 |
| Vaginal delivery, n (%) | 422 (42) | 132 (44) | 290 (41) | 0.442 | 0.506 |
| Amniotic fluid clear, n (%) | 919 (91) | 276 (91) | 643 (91) | 0 | 1 |
| Amniotic fluid volume normal, n (%) | 934 (93) | 280 (92) | 654 (93) | 0 | 1 |

SD, standard deviation; IQR, interquartile range; DR, indicates delivery room; PIH, pregnancy-induced hypertension; RDS, Respiratory distress syndrome; IMV, invasive mechanical ventilation; NIV, noninvasive ventilation; PEEP, positive end expiratory pressure; FiO_2_, fraction of inspired oxygen; SpO_2_, pulse oxygen saturation; NICU, neonatal intensive care unit; RBC, Red blood cell count.

Supplementary-Table 2. Comparison of positive and negative data in the training set

| **Variables** | **Total (n = 706)** | **non-severe IVH**  **(n = 638)** | **severe IVH**  **(n = 68)** | **statistic** | ***P* value** |
| --- | --- | --- | --- | --- | --- |
| **Neonatal characteristics** |  |  |  |  |  |
| Gestational age, wk, mean (SD) | 29.76±2.35 | 30.00±2.23 | 27.45±2.15 | 8.995 | < 0.001 |
| Birth weight, g, mean (SD) | 1174.27±220.80 | 1194.98±208.55 | 980.04±239.00 | 7.961 | < 0.001 |
| Male, n (%) | 374 (53) | 334 (52) | 40 (59) | 0.79 | 0.374 |
| 1-min Apgar score, Median (IQR) | 8 (7, 8) | 8 (8, 8) | 7 (4, 8) | -7.238 | < 0.001 |
| 1-min Apgar score≤7, n (%) | 198 (28) | 153 (24) | 45 (66) | 52.146 | < 0.001 |
| 5-min Apgar score, Median (IQR) | 9 (8, 9) | 9 (9, 9) | 8 (7, 9) | -6.106 | < 0.001 |
| 5-min Apgar score≤7, n (%) | 76 (11) | 55 (9) | 21 (31) | 29.428 | < 0.001 |
| DR resuscitation, n (%) | 170 (24) | 128 (20) | 42 (62) | 56.198 | < 0.001 |
| DR intubation, n (%) | 79 (11) | 53 (8) | 26 (38) | 52.415 | < 0.001 |
| DR chest compression, n (%) | 22 (3) | 15 (2) | 7 (10) | Fisher | 0.003 |
| DR epinephrine, n (%) | 19 (3) | 13 (2) | 6 (9) | Fisher | 0.006 |
| DR surfactant, n (%) | 27 (4) | 22 (3) | 5 (7) | Fisher | 0.17 |
| Transport time after birth, h, Median (IQR) | 0.68 (0.68, 1.68) | 0.68 (0.68, 1.68) | 0.68 (0.68, 5.75) | -2.780 | 0.005 |
| Transport duration, h, Median (IQR) | 0.16 (0.16, 0.16) | 0.16 (0.16, 0.16) | 0.16 (0.16, 0.27) | -2.551 | 0.011 |
| Transport radius, km, Median (IQR) | 3 (3, 3) | 3 (3, 3) | 3 (3, 12.5) | -2.549 | 0.011 |
| IMV during transportation, n (%) | 223 (32) | 172 (27) | 51 (75) | 63.426 | < 0.001 |
| Age on admission, h, Median (IQR) | 1 (1, 2) | 1 (1, 2) | 1 (1, 6.5) | -2.775 | 0.006 |
| Weight on admission, g, mean (SD) | 1164.72±221.77 | 1185.99±209.05 | 965.15±239.37 | 8.161 | < 0.001 |
| RDS on admission, n (%) | 522 (74) | 455 (71) | 67 (99) | 22.224 | < 0.001 |
| NIV on admission, n (%) | 276 (39) | 265 (42) | 11 (16) | 15.549 | < 0.001 |
| IMV on admission, n (%) | 252 (36) | 196 (31) | 56 (82) | 69.138 | < 0.001 |
| Max PEEP, Median (IQR) | 6 (5, 6) | 6 (5, 6) | 6 (6, 6) | -4.869 | < 0.001 |
| Max FiO_2_, Median (IQR) | 30 (25, 40) | 30 (25, 35) | 50 (30, 80) | 8.420 | < 0.001 |
| Max FiO_2_＞30%, n (%) | 484 (69) | 420 (66) | 64 (94) | 21.516 | < 0.001 |
| Max FiO_2_＞40%, n (%) | 186 (26) | 140 (22) | 46 (68) | 63.814 | < 0.001 |
| Max FiO_2_＞60%, n (%) | 64 (9) | 39 (6) | 25 (37) | 66.369 | < 0.001 |
| Lowest SpO_2_ on admission＜90%, n (%) | 146 (21) | 120 (19) | 26 (38) | 12.978 | < 0.001 |
| Lowest SpO_2_ on admission＜85%, n (%) | 54 (8) | 42 (7) | 12 (18) | 9.14 | 0.003 |
| Body temperature on admission＜36.5℃, n (%) | 615 (87) | 330 (52) | 33 (49) | 0.139 | 0.709 |
| Body temperature on admission＜36.0℃, n (%) | 363 (51) | 560 (88) | 55 (81) | 2.022 | 0.155 |
| Hypotension on admission, n (%) | 115 (16) | 95 (15) | 20 (29) | 8.468 | 0.004 |
| Blood glucose on admission＜2.6mmol/L, n (%) | 99 (14) | 86 (13) | 13 (19) | 1.186 | 0.276 |
| Blood glucose on admission＜2.2mmol/L, n (%) | 67 (9) | 56 (9) | 11 (16) | 3.103 | 0.078 |
| Blood glucose on admission＞7.0mmol/L, n (%) | 60 (8) | 48 (8) | 12 (18) | 6.849 | 0.009 |
| Dopamine hydrochloride, n (%) | 239 (34) | 199 (31) | 40 (59) | 19.738 | < 0.001 |
| Dobutamine hydrochloride, n (%) | 315 (45) | 272 (43) | 43 (63) | 9.738 | 0.002 |
| NICU surfactant, n (%) | 356 (50) | 302 (47) | 54 (79) | 24.025 | < 0.001 |
| NICU transfusion, n (%) | 67 (9) | 46 (7) | 21 (31) | 37.382 | < 0.001 |
| Umbilical vein catheterization, n (%) | 259 (37) | 221 (35) | 38 (56) | 11.042 | < 0.001 |
| NICU epinephrine, n (%) | 26 (4) | 13 (2) | 13 (19) | Fisher | < 0.001 |
| NICU caffeine, n (%) | 446 (63) | 394 (62) | 52 (76) | 5.104 | 0.024 |
| RBC on admission＜4.4×10^12^/L, n (%) | 404 (57) | 347 (54) | 57 (84) | 20.565 | < 0.001 |
| Hemoglobin on admission＜114g/L, n (%) | 29 (4) | 19 (3) | 10 (15) | Fisher | < 0.001 |
| Hemoglobin on admission＜100g/L, n (%) | 14 (2) | 8 (1) | 6 (9) | Fisher | 0.001 |
| Hematokrit on admission＜28%, n (%) | 12 (2) | 7 (1) | 5 (7) | Fisher | 0.003 |
| Hematokrit on admission＜45%, n (%) | 159 (23) | 123 (19) | 36 (53) | 38 | < 0.001 |
| Platelet count on admission＜100×10^9^/L, n (%) | 58 (8) | 41 (6) | 17 (25) | 25.705 | < 0.001 |
| Platelet count on admission＜150×10^9^/L, n (%) | 169 (24) | 143 (22) | 26 (38) | 7.602 | 0.006 |
| Total protein on admission＜44g/L, n (%) | 443 (63) | 385 (60) | 58 (85) | 15.314 | < 0.001 |
| Albumin on admission＜32.8g/L, n (%) | 514 (73) | 451 (71) | 63 (93) | 13.875 | < 0.001 |
| Globulin on admission＜8.8g/L, n (%) | 160 (23) | 143 (22) | 17 (25) | 0.11 | 0.74 |
| K^+^＜4.6mmol/L, n (%) | 164 (23) | 151 (24) | 13 (19) | 0.481 | 0.488 |
| K^+^＞6.7mmol/L, n (%) | 36 (5) | 35 (5) | 1 (1) | Fisher | 0.242 |
| Na^+^＜133mmol/L, n (%) | 24 (3) | 19 (3) | 5 (7) | Fisher | 0.071 |
| Na^+^＞146mmol/L, n (%) | 27 (4) | 21 (3) | 6 (9) | Fisher | 0.037 |
| Ca^2+^＜1.53mmol/L, n (%) | 17 (2) | 12 (2) | 5 (7) | Fisher | 0.018 |
| **Maternal characteristics** |  |  |  |  |  |
| Maternal age, y, mean (SD) | 30.80±4.88 | 30.78±4.93 | 31.01±4.45 | -0.381 | 0.703 |
| PIH, n (%) | 147 (21) | 138 (22) | 9 (13) | 2.142 | 0.143 |
| Maternal diabetes, n (%) | 107 (15) | 98 (15) | 9 (13) | 0.082 | 0.774 |
| Maternal heart diseases, n (%) | 13 (2) | 7 (1) | 6 (9) | Fisher | < 0.001 |
| Clinical chorioamnionitis, n (%) | 17 (2.4) | 11 (1.7) | 6 (8.8) | 10.332 | 0.001 |
| Test tube baby, n (%) | 165 (23) | 139 (22) | 26 (38) | 8.388 | 0.004 |
| Multiple births, n (%) | 257 (36) | 225 (35) | 32 (47) | 3.199 | 0.074 |
| Placental abruption, n (%) | 24 (3) | 20 (3) | 4 (6) | Fisher | 0.277 |
| Premature rupture of membranes≥24h, n (%) | 188 (27) | 173 (27) | 15 (22) | 0.566 | 0.452 |
| Fetal distress, n (%) | 45 (6) | 39 (6) | 6 (9) | Fisher | 0.428 |
| Antenatal steroids complete, n (%) | 232 (33) | 213 (33) | 19 (28) | 0.597 | 0.44 |
| Antenatal steroids partial, n (%) | 218 (31) | 205 (32) | 13 (19) | 4.286 | 0.038 |
| Vaginal delivery, n (%) | 290 (41) | 246 (39) | 44 (65) | 16.295 | < 0.001 |
| Amniotic fluid clear, n (%) | 643 (91) | 590 (92) | 53 (78) | 14.236 | < 0.001 |
| Amniotic fluid volume normal, n (%) | 654 (93) | 591 (93) | 63 (93) | 0 | 1 |

SD, standard deviation; IQR, interquartile range; DR, indicates delivery room; PIH, pregnancy-induced hypertension; RDS, Respiratory distress syndrome; IMV, invasive mechanical ventilation; NIV, noninvasive ventilation; PEEP, positive end expiratory pressure; FiO_2_, fraction of inspired oxygen; SpO_2_, pulse oxygen saturation; NICU, neonatal intensive care unit; RBC, Red blood cell count.
